# The mitochondrial genome of *Platencyrtus parkeri* Feriere (Hymenoptera: Encyrtidae)

**DOI:** 10.1080/23802359.2019.1674729

**Published:** 2019-10-11

**Authors:** Yan-Zhou Zhang, Mei Xiong, Qing-Song Zhou, Gong-Cheng Jiang, Chao-Dong Zhu

**Affiliations:** a Jiangsu Key Laboratory of Biofunctional Molecule, School of Life Sciences, Chemistry & Chemical Engineering, Jiangsu Second Normal University, Nanjing, China;; b Key Laboratory of Zoological Systematics and Evolution, Institute of Zoology, Chinese Academy of Sciences, Beijing, China;; c University of Chinese Academy of Sciences (UCAS), Beijing, China

**Keywords:** Mitochondrial genome, Hymenoptera, Encyrtidae, *Platencyrtus parkeri* Feriere

## Abstract

The mitochondrial genome of the *Platencyrtus parkeri* Feriere (Hymenoptera: Encyrtidae) was obtained via next-generation sequencing. The assembled mitogenome is 13,393 bp in length, which contains 33 classical eukaryotic mitochondrial genes with three tRNA genes and rrnS gene missing. All the 13 PCGs begin with typical ATN codons. The 19 detected tRNAs range from 58 to 70 bp in length with typical cloverleaf structure except for trnS1, whose dihydrouridine (DHU) arm forms a simple loop. Meanwhile, they have six tRNAs inserted between nad2 and nad3 compared with *Encyrtus infelix*. Phylogenetic analysis highly supported the monophyly of Pteromalidae. Eupelmidae and Encyrtidae have a close relationship. Within Encyrtidae, *Platencyrtus parkeri* Feriere and *Encyrtus infelix* are close to each other.

*Nipponaclerda biwakoensis* (Kuwana) (Hemeptera: Aclerdidae) is a main injurious pest on *Phragmites australis* in East Asia (Xu and Wang 2010; Noyes 2019) and now is invaded into southern Louisiana, USA (Knight et al. 2018). *Platencyrtus parkeri* Feriere (Hymenoptera: Encyrtidae) is one of the important parasitoids (Xu and Wang 2010; Noyes 2019), which show high host specificity to *N. biwakoensis* (unpublished data). As a gregarious endoparasitoid of *N. biwakoensis*, *Platencyrtus parkeri* might be very useful to biocontrol the population density of *Nipponaclerda biwakoensis* in the future. So far as we know, little knowledge on their genetic information is available. Here, we present the mitochondrial genome of *P. parkeri* Feriere.

Specimen of *P. parkeri* Feriere (Voucher number: XM19002) was reared from *N. biwakoensis* collected in Ningbo, Zhejiang. Voucher specimens of this study were deposited in the Institute of Zoology, Chinese Academy of Sciences (IZCAS). The total mitochondrial genome of *P. parkeri* Feriere was obtained through next-generation sequencing. The extracted DNA mixture were applied for library construction by the usage of Illumina TruSeq@ DNA PCR-Free HT Kit, and sequenced by the platform of Ilumina HiSeq sequencer (150 bp pared-end). The mitochondrial genome of *P. parkeri* Feriere was assembled based on Illumina short reads with NOVOPlasy v2.7.0 (Dierckxsens et al. 2017) using COI sequence as the initial seed. The whole mitochondrial genome annotation was annotated by Mitos WebServer (http://mitos2.bioinf.uni-leipzig.de/index.py) under the invertebrate mitochondrial code (Bernt et al. 2013). Transfer RNA (tRNA) genes were confirmed by online ARWEN (http://130.235.46.10/ARWEN/) (Laslett and Canback 2008). The GenBank accession number of *P. parkeri* is MN296710.

The mitogenome sequence of *P. parkeri* Feriere was 13,393 bp in length with A + T content of 82.3%. It consists of 13 protein-coding genes (PCGs), 19 transfer RNAs (tRNAs), and one partial 16 ribosomal RNAs (rRNAs). Three tRNAs, 12S rRNA, and control region were missed. All 13 PCGs were initiated by typical ATN codons (eight ATT and five ATG). Ten genes use TAA as terminal stop, one gene stop with TAG, two genes have incomplete stop codon. All of the 19 tRNA genes, ranging from 58 to 70 bp, have a typical cloverleaf structure except for trnS1, whose dihydrouridine (DHU) arm forms a simple loop. The absence of the DHU arm in trnS1 was found in the mitochondrial genomes existed in most insects (Wolstenholme 1992). The rrnL genes is 837 bp, with an average A + T content of 85.2%. Additionally, 22 intergenic spacers (234 bp in total) and 5 overlapping regions (19 bp in total) are dispersed throughout the genome. The inversion of six PCGs (including nad3, cox3, atp6, atp8, cox2 and cox1) has also been found in *P. parkeri* which consisted of other chalcidoids (Oliveira et al. 2008). Besides, they also have six tRNAs inserted between nad2 and nad3 compared with *Encyrtus infelix* (Xiong et al. 2019).

The mitogenomic sequences of 22 chalcidoid species were used to reconstruct the phylogeny of Chalcidoidea. Two species from superfamily Proctotrupoidea (*Vanhornia eucnemidarum* and *Pelecinus polyturator*) were chosen as outgroup. Phylogenetic analyses based on 13 PCGs were incomplete PCGs in some species that were identified using MrBayes (Ronquist et al. 2012). The nodes of bayesian inference phylogeny tree with high support value are shown in Figure 1. Generally, Mymaridae was always at the basal position within Chalcidoidea (Sharkey et al. 2012; Heraty et al. 2013). The monophyly of Encyrtidae was strongly supported, showing a sister relationship with Eupelmidae (Xiong et al. 2019).

**Figure 1.**
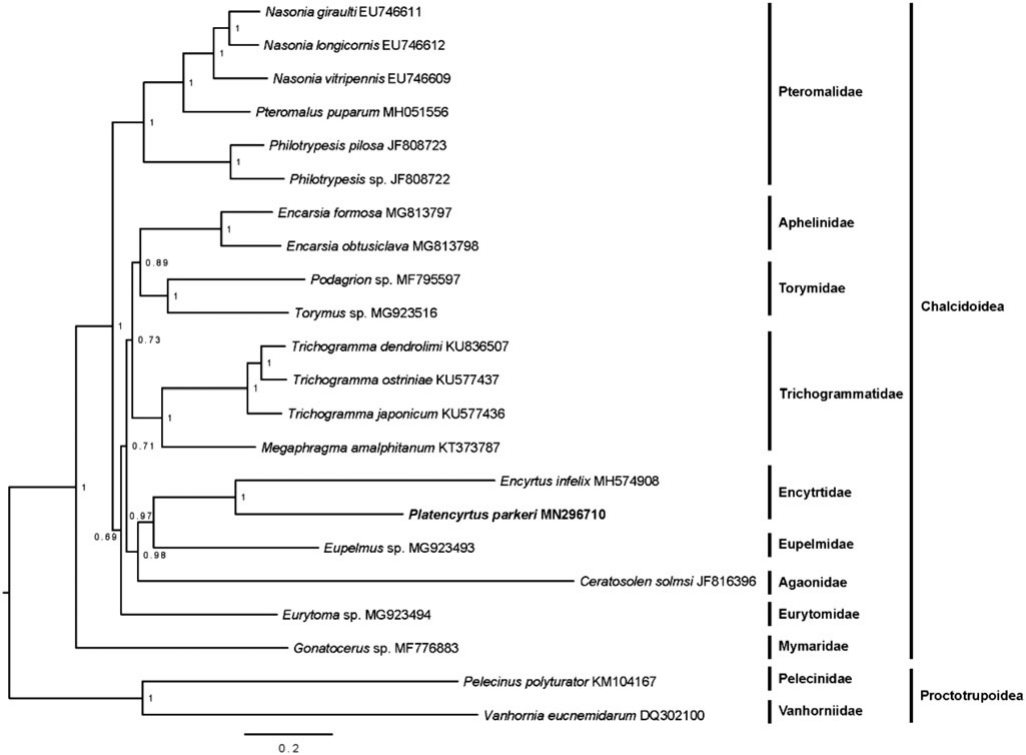
The molecular phylogeny of Chalcidoidea based on 13 PCGs. The phylogenetic tree was constructed by Bayesian inference. Each species involved in the tree has scientific name on the right side.
